# Comparison of artificial intelligence assisted training and traditional learning paths in clinical simulation skills training: meta-analysis of randomized controlled trials

**DOI:** 10.3389/fmed.2026.1915528

**Published:** 2026-08-21

**Authors:** Sihan Che, Huan Zhao, Tianlin Lu, Zhen You

**Affiliations:** 1 Division of Biliary Surgery, Department of General Surgery, West China Hospital, Sichuan University, Chengdu, Sichuan, China; 2 Administrative Office, West China School of Medicine, Sichuan University, Chengdu, Sichuan, China; 3 Academic Affairs Department, West China School of Medicine, Sichuan University, Chengdu, Sichuan, China

**Keywords:** artificial intelligence, clinical skill, medical education, simulation, traditional learning

## Abstract

**Background:**

Artificial intelligence (AI) is increasingly integrated into clinical simulation training, yet its overall effectiveness compared to traditional learning remains unclear. This meta-analysis of randomized controlled trials (RCTs) aimed to evaluate the impact of AI-assisted training versus traditional instruction on clinical skills performance, procedure time, and learner satisfaction.

**Methods:**

PubMed, Embase, and Web of Science were searched from inception to April 10, 2026. RCTs comparing AI-assisted training (real-time feedback, intelligent tutoring, AI-simulated patients, or image recognition support) with traditional instruction (expert-led, self-directed, or role-play) in simulation-based clinical skills training were included. The primary outcome was clinical skills score. Standardized mean differences (SMD) with 95% confidence intervals (CI) were calculated using fixed- or random-effects models based on heterogeneity.

**Results:**

Twelve RCTs were included. Overall, significant difference was found between AI-assisted and traditional training for clinical skills score. Subgroup analysis revealed that AI significantly improved surgical and invasive procedural skills, but showed no advantage for clinical assessment and diagnostic skills. In addition, the vision-based deep-learning AI model may have more advantages in medical training than the traditional training method, while the large language model did not demonstrate statistically significant differences with the traditional training method. Procedure time and Operation completion rate did not differ between groups. Learner satisfaction and willingness to recommend the training and stress level of AI assisted training were higher than those of the control group.

**Conclusion:**

This study shows that AI, especially the vision-based deep-learning, can effectively guide the training of procedural skills. However, for complex cognitive skills like clinical reasoning, AI is best used to replace inefficient peer practice with standardized, high-intensity training. Expert teachers should then focus on advanced clinical judgment, emotional communication, and personalized correction. Overall, AI offers a major opportunity to improve quality and scale up medical simulation education. Its best path is deep integration with traditional teaching—combining their strengths rather than simply replacing teachers.

**Systematic review registration:**

https://www.crd.york.ac.uk/PROSPERO/view/CRD420261411402. The protocol was registered in advance in PROSPERO online platform as CRD420261411402.

## 1 Introduction

The proficiency of clinical skills directly impacts the quality of medical care and patient safety. Whether it involves invasive procedures such as bronchoscopy and laparoscopy, or assessment skills like history taking, doctor-patient communication, and ultrasound image interpretation, efficient skill acquisition and continuous improvement are the goals of clinical competency training (1–3). However, traditional training models have long faced a theory–practice gap, insufficient personalized and immediate feedback, and a heavy reliance on expert instructors for teaching resources, making it difficult to meet the demands of large-scale, standardized, and efficient teaching (4). While simulation training provides learners with a risk-free, repeatable practice environment, the quality and consistency of feedback still largely depend on the experience and time commitment of instructors.

In recent years, the rapid development of artificial intelligence (AI) has offered new possibilities to overcome the aforementioned challenges (5). In medical simulation training, AI-assisted training systems primarily manifest in the following forms: real-time operational feedback systems (using computer vision or sensor data to automatically identify anatomical structures, assess operational risks, prompt correct steps, and provide visual progress tracking during the learner’s performance); intelligent tutoring systems (using machine learning algorithms to perform multi-level analysis of learner’s operational data, such as safety indicators and instrument movement efficiency, and provide phased guidance); large language model-driven simulated patients (capable of engaging in natural language conversations with learners, simulating real clinical interview scenarios, and providing structured feedback reports after the interview); and image recognition assistance systems (helping learners identify key anatomical structures in image-dependent skills such as ultrasound and endoscopy, and optimize image acquisition quality) (6, 7). The common advantages of these systems are immediacy, standardization, repeatability, and quantifiability.

Currently, RCTs have compared AI-assisted training with traditional learning methods across different clinical skill domains, including bronchoscopy, laparoscopic surgery, ultrasound diagnosis, doctor-patient communication, and history taking. However, evidence directly comparing AI’s impact on procedural versus cognitive skills within a single analytical framework, or distinguishing between vision-based and language-based AI systems, remains limited (8). Therefore, we conducted this meta-analysis to synthesize available RCT evidence, with subgroup analyses stratified by skill type and AI technology, aiming to provide evidence-based guidance for integrating AI into clinical simulation curricula. The primary outcome measure was the comprehensive clinical skills score, while secondary outcomes included procedure time, operation completion rate, learner satisfaction/recommendation willingness and stress level.

## 2 Materials and methods

We systematically searched PubMed, Embase, and Web of Science for studies published from the inception of each database until April 10, 2026, that met the predefined eligibility criteria. The search strategy incorporated the following keywords: “artificial intelligence” OR “machine learning” OR “deep learning” OR “AI assisted” OR “AI guided” OR “intelligent tutoring,” combined with “simulation” OR “simulator” OR “virtual reality” OR “manikin,” as well as “technical skill” OR “procedural skill” OR “surgical skill” OR “operative performance.” Additionally, we manually screened the reference lists of relevant articles and previous systematic reviews to identify additional potentially eligible studies. Only RCTs were included. The systematic review and meta-analysis were conducted in accordance with the guidelines set forth by the Cochrane Collaboration, and the results were reported in adherence to the PRISMA (Preferred Reporting Items for Systematic Reviews and Meta-Analyses) reporting guidelines.

### 2.1 Inclusion criteria

(1) Study design: randomized controlled trials; (2) Population: medical students, residents, and nursing students undergoing simulation-based clinical skills training; (3) Intervention: experimental group received artificial intelligence (AI)-assisted training (including but not limited to real-time AI feedback, post-hoc AI feedback, AI-augmented human instruction, AI-based simulated patients, AI chatbots, etc.); (4) Comparison: control group received traditional instruction (including expert/instructor-led training, self-directed learning, written materials, role-play, standardized patients, etc.); (5) Outcomes: at least one quantitative outcome related to clinical skills (e.g., procedure time, global technical skill score, diagnostic completeness, OSCE score, history-taking ability, communication skill score, etc.). For each included study, we documented whether the assessment tool used was previously validated or standardized; studies were not excluded solely based on the lack of formal validation, but this information was recorded and considered in the interpretation of findings; (6) Full-text availability.

### 2.2 Exclusion criteria

(1) Full-text cannot be obtained; (2) No control group, inadequate control group (e.g., historical control without concurrent comparison), insufficient trial data, or poor data quality (e.g., lack of reported means, standard deviations, or sample sizes for key outcomes); (3) Publication types including case reports, reviews (systematic or narrative), conference abstracts/reports, letters, comments, editorials, or animal experiments; (4) AI used only for diagnostic or image recognition purposes without providing educational feedback, unless the study includes a training comparison group that meets the inclusion criteria; (5) Outcomes not relevant to clinical skill performance (e.g., only satisfaction, motivation, or cognitive load without any performance metric); (6) Non-English language publications.

### 2.3 Data extraction and literature quality evaluation

**Figure 1:**
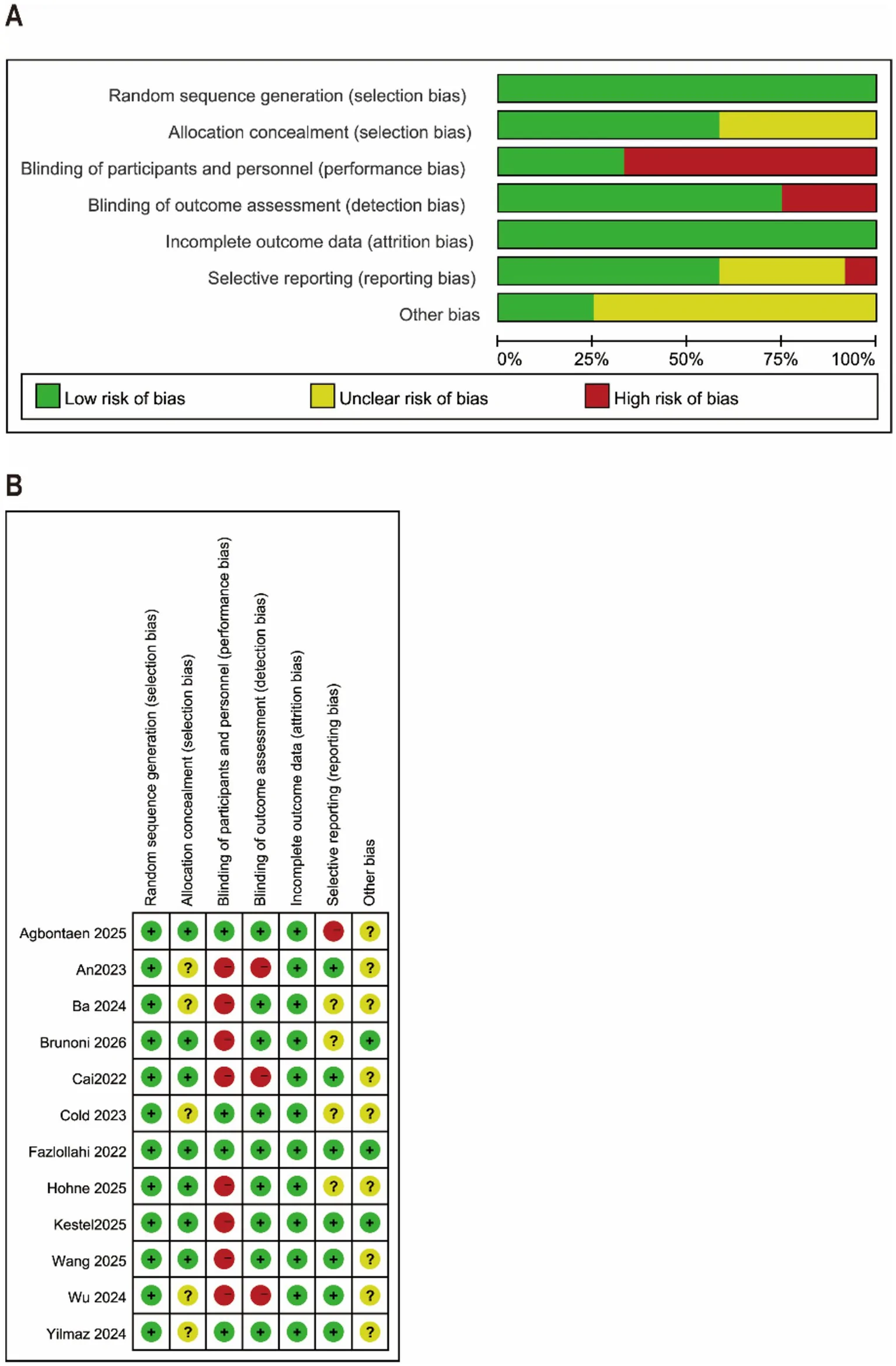
Risk of bias assessment. **(A)** Risk of bias across all studies. **(B)** Risk of bias for each item in each study.

Two reviewers independently extracted the following parameters from each study: first author, publication year, country, surgical/procedural domain, study design, sample size per group, type of AI intervention, type of control intervention, and outcome measures. Any discrepancies between the reviewers were resolved through consensus. The 12 included RCTs comprised a total of 546 participants. The quality of the included randomized controlled trials was assessed using the Cochrane Risk of Bias tool (RoB), which evaluates seven domains: Random sequence generation (selection bias), Allocation concealment (selection bias), Blinding of participants and personnel (performance bias), Blinding of outcome assessment (detection bias), Incomplete outcome data (attrition bias), Selective reporting (reporting bias), Other bias. Each domain was judged as “Low Risk,” “Unclear Risk” or “High Risk” (Figure 1).

### 2.4 Statistical analyses

Statistical analysis of outcome measures was performed using Review Manager 5.4 software. SMD with 95% confidence interval (CI) was used as the effect measure for continuous outcomes, as different studies used different scales to assess technical skills (e.g., ICEMS, OSATS, LCRF, BSTAT) or measured procedure time in different surgical contexts, etc. A fixed-effects model was applied when heterogeneity was low (I^2^ ≤ 50% and *p* > 0.1). In cases of substantial heterogeneity (I^2^ > 50% and *p* < 0.1), a random-effects model was employed, followed by subgroup or sensitivity analyses to explore potential sources of heterogeneity. The results are presented using forest plots. When the number of included studies was ≥10, funnel plot assessment and Egger’s test were used to analyze the potential bias. In the Overall Clinical Skills Score section, subgroup analyses were conducted based on skill types and AI model types, with the subgroup analysis criteria being: (1) Procedural/invasive skills: These refer to operative techniques that involve the entry of endoscopes, puncture needles, or surgical instruments into body cavities or tissues. The core learning objectives are proficiency in instrument manipulation and procedural safety. Assessment is primarily based on objective parameters, such as step completion rates, procedure time, and other quantifiable metrics. Clinical assessment/diagnostic skills: These refer to non-invasive clinical tasks centered on information gathering, data synthesis, and clinical reasoning. They do not involve instrument entry into body cavities or tissue puncture. Assessment relies mainly on subjective scoring methods, including OSCEs, completeness of history-taking, communication skills, and similar qualitative evaluations. (2) Vision-based AI model: Based on deep learning or convolutional neural networks, it can perform anatomical structure recognition, operation trajectory monitoring, and real-time feedback on images/videos. Large language model: Based on a generative pre trained Transformer architecture, it guides medical history collection and clinical reasoning feedback through natural language dialog.

### 2.5 Grading the evidence

We use the Grading of Recommendations Assessment, Development and Evaluation (GRADE) method to determine the certainty of evidence (9). In the GRADE tool, randomized controlled trials start with high-level evidence, and the evaluation process may be compromised by five factors: publication bias, inconsistencies, indirect evidence, imprecision, and methodological limitations. At the end of the analysis, the GRADE system rated the level of evidence in the meta-analysis as high, moderate, low, or very low (10).

## 3 Results

### 3.1 Study selection

**Figure 2:**
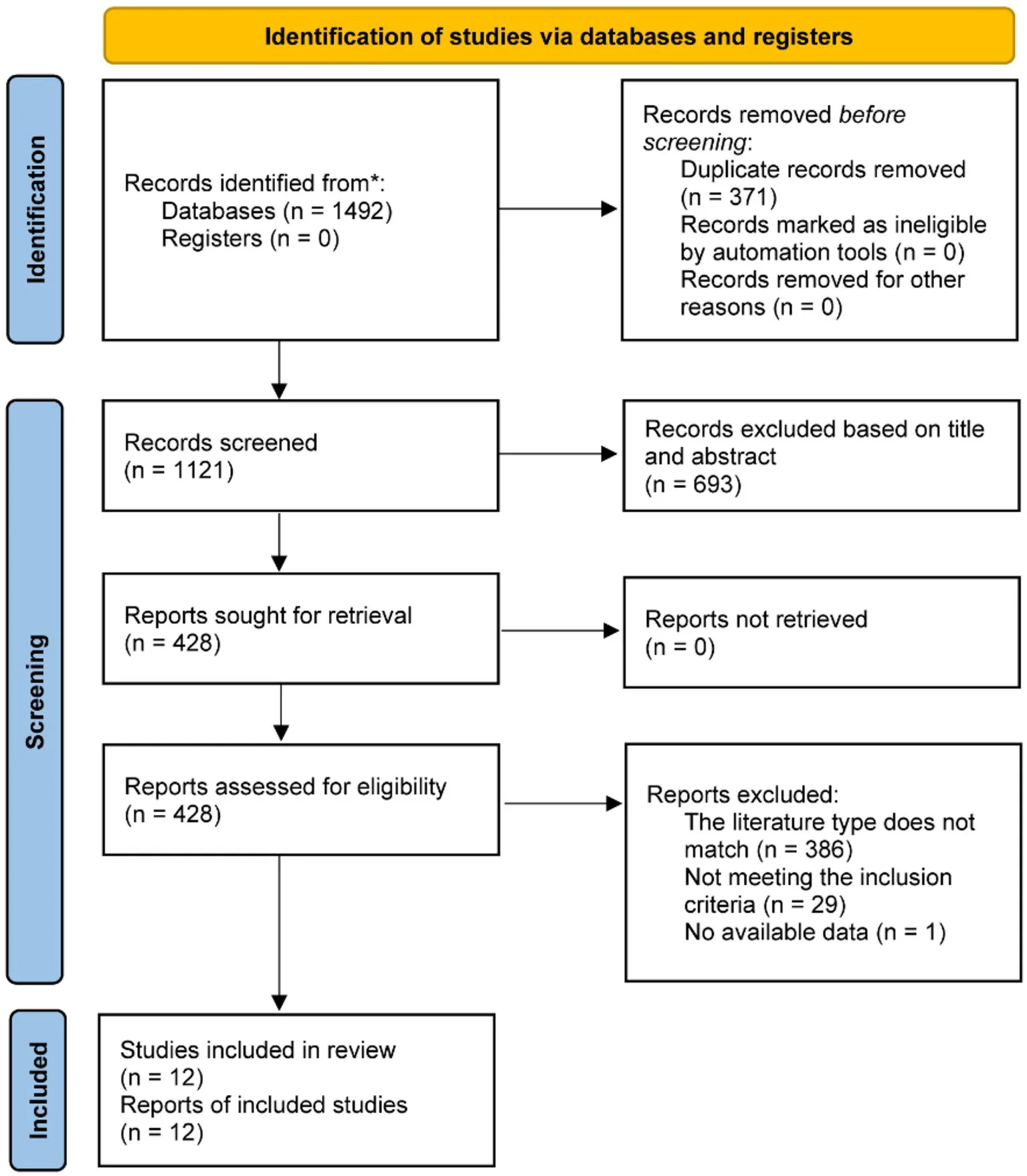
Flow chart showing the study selection process (in accordance with the PRISMA flow diagram).

This process led to the exclusion of studies that did not meet the inclusion criteria, resulting in 12 eligible articles (11–22), all included studies were RCT in design. The literature selection process is illustrated in Figure 2, and the basic characteristics of the included studies are presented in Table 1.

**Table 1:** Characteristics of the studies included in the meta-analysis.

Author	Year	Population	Intervention	Comparison	Ref.
Cold	2023	Medical students with no prior bronchoscopy experience	Training using an AI-based automatic bronchial segment identification system, providing real-time on-screen labels, lung tree diagram, structured progress score, etc.	Traditional written instruction with only procedure time feedback, no AI assistance	(11)
Wu	2024	Novice surgeons	After each procedure, AI system automatically generates quantitative surgical metrics; coach and trainee discuss based on AI report and intelligent visualization platform during online coaching sessions	Independent self-learning after each procedure	(12)
Yilmaz	2024	Medical trainees (years 1–4) with no prior experience using the NeuroVR surgical simulator	Real-time auditory instructions from ICEMS during practice tasks, followed by AI-generated video clips of errors after each task	Real-time verbal instruction and post-task feedback provided in-person by a neurosurgery resident	(13)
Fazlollahi	2022	Medical students (preparatory, 1st, or 2nd-year) with no surgical experience and no prior use of the NeuroVR simulator	AI tutor group: quantitative, metric-based audiovisual feedback with a stepwise learning approach (first achieve expert level on safety metrics, then instrument movement metrics)	Remote expert instruction: standardized synchronous verbal feedback and guidance provided by a neurosurgery resident via remote session	(14)
Agbontaen	2025	Critical care physicians who had performed at least one unsupervised bronchoscopy in a mechanically ventilated ICU patient	30 min of self-directed training using an AI system (Ambu Broncho Simulator)	30 min of one-on-one bedside instruction by two experienced pulmonary/critical care consultants, without AI feedback	(15)
Brunoni	2026	Second-year anesthesia and intensive care residents with little or no bronchoscopy experience	20 min of unsupervised training using an AI-based image recognition software	20 min of one-on-one traditional training by an experienced bronchoscopy expert	(16)
Ba	2024	Pediatric trainee (5-year medical intern)	ChatGPT assisted teaching (using ChatGPT 4.0 version, combined with case interaction, real-time feedback, bedside practice, etc.)	Traditional teaching methods (standard bedside teaching, including case demonstrations, student independent operations, feedback discussions, etc.)	(17)
Höhne	2025	Clinical stage medical students	AI supported self-learning method (using ScanLab application, including AI real-time anatomical structure labeling, image quality feedback, etc.)	Traditional hands-on teaching (mentor on-site guidance on operating handheld ultrasound equipment, image acquisition and interpretation)	(18)
Wang	2025	Fifth year medical student (5th-year, before clinical rotation), without experience in simulating patients or structured medical history collection training	GPT simulation patient (customized development based on ChatGPT GPT platform), students and AI conduct 30 min/time, 3 times a week, for a total of 4 weeks of medical history collection simulation training, with AI providing automatic feedback	Traditional role-playing (played by experienced clinical skill instructors as patients for the same duration and frequency, providing structured feedback)	(19)
An	2023	32 graduate students majoring in gastroenterology with no relevant experience	AIDRS assisted gastroscopy training: real time monitoring of blind area of gastroscopy, assisting in lesion identification and diagnosis, providing operation score and risk prompt	Routine gastroscopy operation training	(20)
Cai	2022	Residents (receiving standardized training), previous experience of ultrasound-guided sciatic nerve block <5 cases	use the AI assisted neural recognition system based on convolutional neural network for training, manually mark the nerves on the iPad and compare with the AI recognition results to obtain score feedback, and Practice on the simulator after reaching the standard	ultrasound operation practice is conducted under the guidance of the instructor on the simulator without AI assisted feedback	(21)
Kestel	2025	A total of 82 1st-year nursing students were randomly divided into two groups	Traditional teaching + AI chat robot (based on unity engine + decision tree algorithm, it simulates virtual patients, provides real-time feedback, and can carry out 2-week asynchronous exercises anytime, anywhere)	Only accept theoretical teaching + video viewing + clinical practice, without chat robot assistance	(22)

### 3.2 Outcomes

#### 3.2.1 Overall clinical skills score

**Figure 3:**
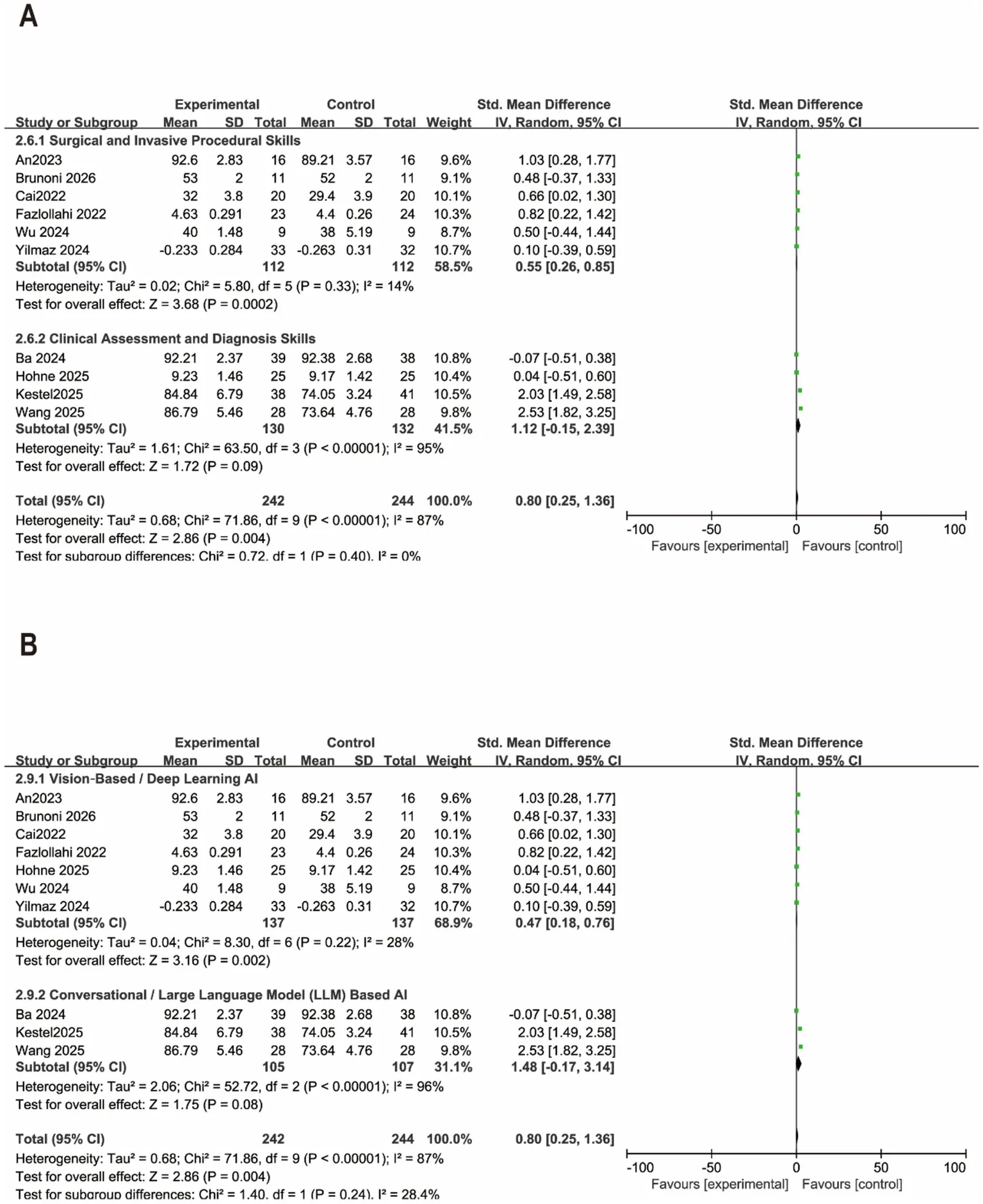
The analyses of overall clinical skills score with the indicator of SMD with 95% CI: **(A)** Training type subgroup; **(B)** AI type subgroup.

A total of 10 studies reported skills scores from clinical simulation training, and subgroup analyses were performed based on different types of experimental groups (Figure 3). Due to differences in scoring schemes across some studies, the SMD was calculated as the effect size. Heterogeneity among the 10 included studies was substantial (I^2^ = 87%), and a random-effects model was adopted. The overall results showed that the overall skill scores of AI assisted instruction and traditional learning methods were significantly different (SMD = 0.80, 95% CI: 0.25 to 1.36, *p* = 0.004). Egger’s test indicated no significant publication bias (*p* = 0.301).

##### 3.2.1.1 Invasive operation skills and clinical evaluation skills

Subgroup analysis for Surgical and Invasive procedural skills indicated that the AI-guided teaching group scored 43% higher than the traditional learning group (SMD = 0.55, 95% CI: 0.26 to 0.85, *p* = 0.0002), with low heterogeneity among studies (I^2^ = 14%). For Clinical assessment and diagnosis skills, the subgroup analysis showed no statistically significant difference in scores between the AI-guided group and the traditional teaching group (*p* = 0.09).

##### 3.2.1.2 Visual AI model and large language AI model

In the subgroup analysis by AI modality, vision-based/deep-learning AI significantly improved skill scores compared to traditional training (SMD = 0.47, *p* = 0.002), with low heterogeneity (I^2^ = 28%). For the subgroup analysis of the large language model, there was no significant difference in the scores between the AI Guidance Group and the traditional teaching group (*p* = 0.08).

#### 3.2.2 Secondary outcomes

**Figure 4:**
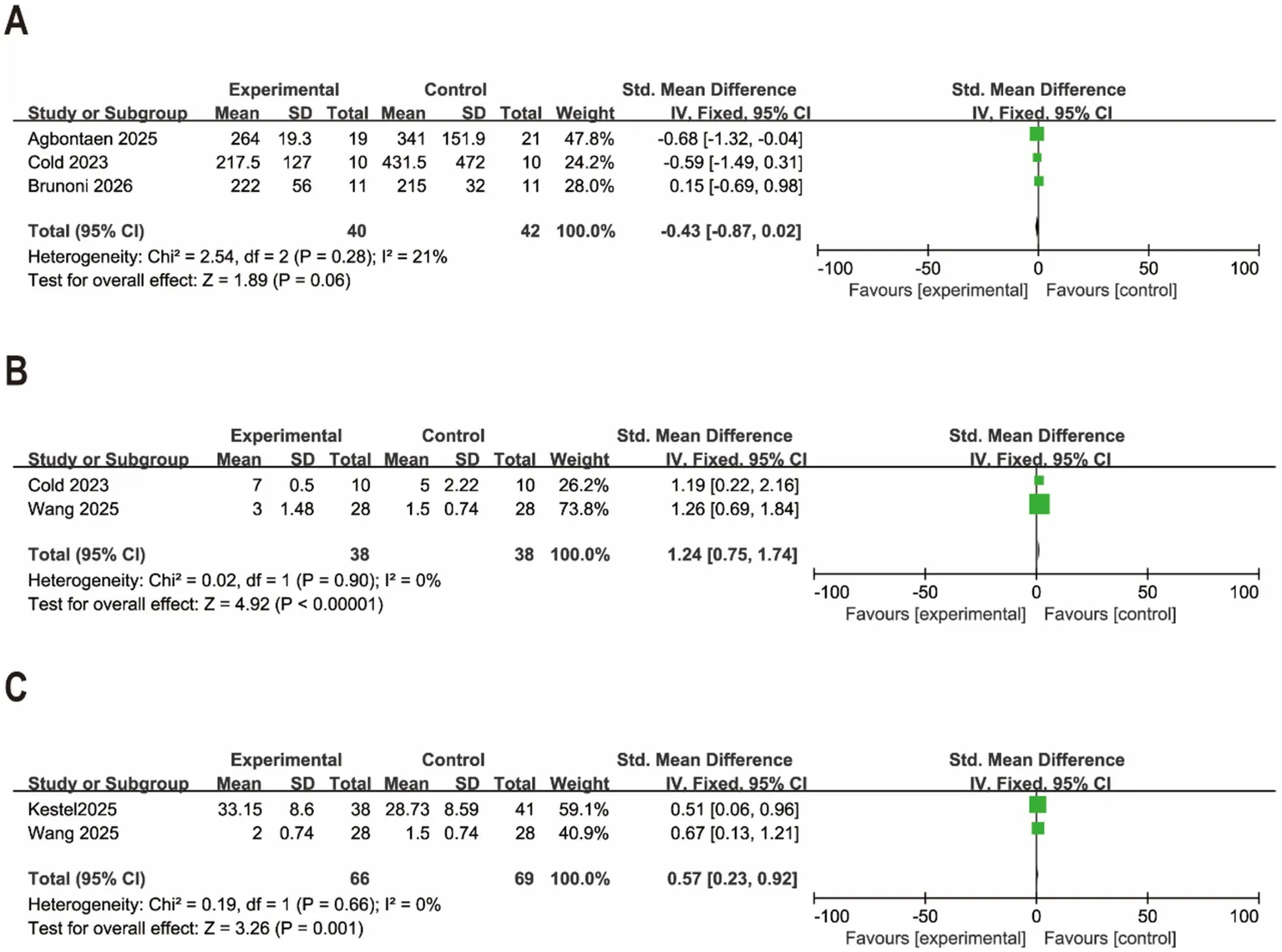
Forest plots of secondary outcomes comparing AI-assisted training with traditional training. **(A)** Procedure time; **(B)** Learner willingness to recommend the training; **(C)** Stress level. Effect sizes are expressed as SMD with 95% confidence intervals.

Procedure time showed a trend toward being shorter in the AI-assisted group, but the difference did not reach statistical significance (SMD = −0.43, 95% CI: −0.87 to 0.02, *p* = 0.06; I^2^ = 21%; Figure 4A). For recommendation outcomes, Cold et al. used the validated Intrinsic Motivation Inventory (IMI, 7-point Likert scale), while Wang et al. used a self-designed questionnaire (5-point Likert scale). Both instruments assessed willingness to recommend the training with consistent directionality. Learners in the AI-assisted group reported significantly higher willingness to recommend the training compared with the traditional group (SMD = 1.24, 95% CI: 0.75–1.74, *p* < 0.0001; I^2^ = 0%; Figure 4B). For stress outcomes, Kestel et al. used the Clinical Stress Questionnaire (CSQ, total score 0–80, higher scores indicating greater stress), while Wang et al. used a single-item self-rating scale (1–5, higher scores indicating greater anxiety). Stress levels were significantly higher in the AI-assisted group than in the traditional group (SMD = 0.57, 95% CI: 0.23–0.92, *p* = 0.001; I^2^ = 0%; Figure 4C).

### 3.3 Publication bias and sensitivity analyses

**Figure 5:**
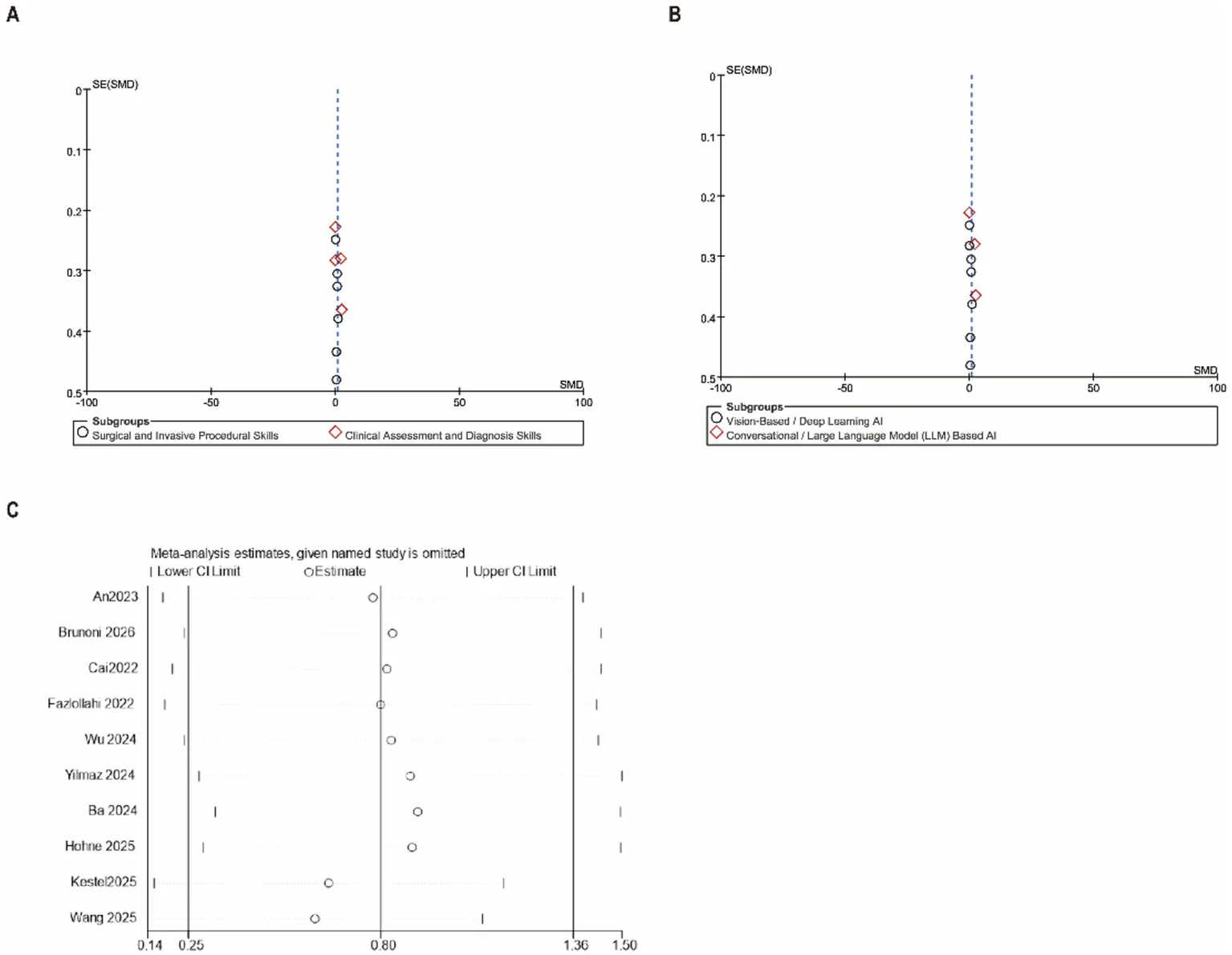
Publication bias and sensitivity analysis for the overall clinical skills score. **(A,B)** Funnel plots for the subgroup analyses by training type **(A)** and AI modality **(B)**. **(C)** Leave-one-out sensitivity analysis showing the influence of each individual study on the pooled effect estimate.

We assessed publication bias for all eligible studies using funnel plot analysis (Figures 5A,B) and Egger’s test. The results indicated no significant publication bias among the included studies. Sensitivity analysis was performed using Stata 16.0 to evaluate whether individual studies disproportionately influenced the overall results. The analysis demonstrated that no single study significantly affected the pooled results (Figure 5C), confirming the robustness of our findings.

### 3.4 Grading the evidence

The certainty of evidence is evaluated using the GRADE method. The certainty of evidence was rated as moderate in Surgical and Invasive Procedural Skills, but was downgraded due to severe imprecision. The evidence certainty ratings for Satisfaction, Stress Level, and Procedure Time are relatively low; The Clinical Assessment and Diagnosis Skills have been downgraded due to significant risks of bias, inconsistency, and inaccuracy, resulting in a very low rating (Supplementary Table S1).

## 4 Discussion

This meta-analysis included 12 RCTs covering multiple clinical simulation training domains, including surgical/invasive procedures and clinical assessment/diagnosis. The main results showed that in the surgical/invasive procedural skills subgroup, AI-assisted training was significantly superior to traditional teaching; in the clinical assessment and diagnosis skills subgroup, there was no statistically significant difference between AI and traditional teaching. In addition, vision-based deep-learning currently performs better in medical training compared to large language models. Learners’ satisfaction/recommendation willingness and stress level for AI-assisted training was higher than for traditional learning. Regarding procedure time and operation completion rate, there was no significant difference between the AI and traditional groups.

The results of this study demonstrate the excellent performance of AI-assisted training in invasive procedural skills training. Escobar-Castillejos et al. (23) also indicated that the application of AI in invasive procedure training shows potential for enhancing skill acquisition and enabling more efficient learning pathways. The studies related to invasive procedures included in this meta-analysis employed various forms of deep-learning AI systems, including phased intelligent tutoring systems, real-time multi-indicator monitoring systems, post-procedure visual reporting systems, and deep learning-based real-time image recognition systems. Although the specific technical pathways differ, they collectively point to a common conclusion: in structured, procedural skills training, AI systems can effectively enhance learners’ operational performance, safety awareness, and efficiency. From the perspective of feedback mechanisms, AI systems achieve immediacy, objectivity, and freedom from judgmental pressure. In traditional teaching, learners must wait for expert feedback after completing a procedure, during which incorrect actions may be reinforced repeatedly without timely correction, forming bad habits. In contrast, AI systems can provide immediate feedback through visual changes or voice prompts the moment an error occurs. This short “action-feedback-adjustment” loop enables learners to make corrections early during the formation of procedural memory, greatly accelerating the consolidation of correct action patterns. In contrast to human instruction, which inherently involves subjective judgment and interpersonal dynamics, AI feedback operates according to unified algorithmic standards and provides consistent, objective evaluations. This distinction does not diminish the value of human teaching—indeed, experienced instructors offer nuanced guidance, emotional support, and adaptive responses that AI cannot replicate—but it does highlight a complementary strength of AI-assisted training. At the same time, as an objective teaching tool, AI does not display negative emotions such as disappointment or impatience, allowing learners to practice trial-and-error without psychological burden. Cold et al. (11) found that the total score on the Intrinsic Motivation Inventory was significantly higher in the AI group than in the control group, Yilmaz et al. (13) also found that the satisfaction levels in the AI group were higher than those in the traditional group, both confirming the positive effect of this pleasant environment on learning motivation. Furthermore, the highly structured nature of surgical procedures aligns well with AI’s technical strengths. Whether it is the sequential exploration of bronchial trees, achieving the critical view of safety in laparoscopic cholecystectomy, or controlling bleeding risk during brain tumor resection, these procedures are essentially rule-based, step-by-step tasks. Kinoshita et al. (24) developed a deep learning model based on U-Net, demonstrating AI’s powerful ability to identify complex anatomical structures intraoperatively. AI systems excel precisely at recognizing, monitoring, and providing feedback on such well-defined behavioral sequences. The more structured the task and the clearer the rules, the more prominent the advantages of AI become. This also explains, from the opposite perspective, why AI’s advantages are less evident in open-ended communication or assessment tasks, which rely on abilities such as empathy and subjective recognition that are difficult to quantify and currently fall outside the expertise of existing AI systems. The studies related to clinical assessment and diagnosis skills included in this meta-analysis exhibited high heterogeneity. An in-depth analysis revealed that the core source of heterogeneity lies in the discrepancy between the intervention and the outcome evaluation. The study by Wang et al. (19) focused on skills training in history taking, clinical reasoning, and doctor-patient communication. These skills are highly dependent on repetitive language interactions, immediate feedback, and flexible responses to clinical scenarios. Compared with traditional role-playing, AI (GPT as a simulated patient) can provide judgment-free, repeatable conversational practice and generate personalized, structured feedback after each interaction. This immediate feedback learning model precisely addresses the inherent shortcomings of traditional role-playing, such as varying peer abilities, delayed or absent feedback, and limited scenarios. In contrast, human instructors played a crucial role in the teaching process in the studies by Ba and Höhne (17, 18). In Ba et al.’s study, both groups shared clinical practice and learning resources, with ChatGPT serving as an auxiliary discussion tool rather than dominating the skills training. Höhne et al.’s evaluation of ultrasound assessment and diagnostic skills relied on instructors providing real-time corrections for operational details and image interpretation. Although AI can provide visual cues such as image quality assessment and anatomical landmark identification, it cannot simulate an instructor’s targeted corrections for incorrect operations or image misinterpretations. Therefore, in these two types of skills, AI did not surpass traditional teaching. Consistent with this pattern, a recent RCT among rehabilitation students found that AI-generated clinical scenarios improved digital competence and attitudes toward AI, but did not yield significant between-group differences in clinical self-efficacy compared with internet-supported or traditional methods (6). This further supports the notion that for complex, open-ended cognitive tasks—such as clinical reasoning and treatment planning—AI’s added value over conventional approaches remains uncertain. Within the LLM subgroup, heterogeneity was extremely high, primarily driven by the study of Ba et al. In that study, ChatGPT served merely as an auxiliary discussion tool, with instructors leading the feedback and correction process and the AI providing no automated evaluation. By contrast, Wang et al. employed GPT as a simulated patient that generated structured four-dimensional rubric-based feedback covering history completeness, clinical reasoning, communication, and professional behavior after each interaction, while Kestel et al. used a chatbot that provided real-time omission prompts during the conversation. The three studies differed substantially in AI role, feedback timing, training dose, and instructor involvement, forming a clear gradient that aligned with the effect size ranking. This gradient suggests that the effectiveness of LLM-assisted training is highly dependent on its functional positioning and instructional design. This also highlights the irreplaceable advantages of instructor-led traditional teaching. Traditional classroom lectures and clinical rotations remain the core pathways for medical students to acquire knowledge. AI can currently only serve as a supplementary tool for simulated discussions or question-answering and cannot replace the systematic construction of knowledge frameworks. Instructors help students form correct habits through on-site observation of their operations and feedback. Experienced teachers can dynamically adjust the teaching pace, supplement cases, or pose reflective questions based on students’ real-time performance, and can also provide genuine emotional support (25, 26). Current AI tools still rely on predefined prompts and fixed feedback templates, lacking genuine pedagogical intelligence. The ability to ask immediate follow-up questions, challenge students, or guide them toward self-correction, which is common in traditional teaching, remains something AI is unlikely to achieve in the short term.

Therefore, it must be emphasized that AI guidance is currently primarily a complementary educational tool, not a replacement for traditional medical education methods. Although this meta-analysis shows that AI-assisted training is superior to traditional teaching in surgical or invasive procedural skills and that learner satisfaction is generally high, the included studies reveal that current AI guidance still has multiple limitations in clinical skills training. First, AI feedback systems have significant blind spots, particularly in guiding non-quantifiable skills. In procedural skills, existing AI systems mainly focus on quantifiable dimensions such as anatomical structure identification, sequence of steps, instrument force, and risk indicators. However, they cannot provide effective feedback on skills that rely on tactile sense and experiential judgment, such as the feel of instrument manipulation in bronchoscopy, avoiding collisions with airway walls, or the fine adjustment of probe pressure and angle in ultrasound examinations. Second, while immediate AI feedback helps beginners correct errors quickly, a study by Fazlollahi et al. (14) found that when AI feedback was turned off, the improvement in learners’ performance on real tasks was limited, suggesting a potential dependence on AI prompts that may not translate into the learner’s own independent judgment ability. An observational study by Budzyń et al. (27) also showed that endoscopists, an artificial intelligence tool, might be at risk of skill decay after continuous exposure to it. Our study reveals that AI-assisted training, particularly in the domain of conversational and history-taking skills, is associated with higher self-reported stress and anxiety levels among learners compared to traditional instruction. This elevation appears to stem from immediate and unfiltered evaluative feedback, the absence of non-verbal social cues in human–AI interaction, increased cognitive processing demands, and heightened self-expectations triggered by novel technology. Crucially, this stress response coexists with superior skill acquisition, suggesting it may represent a form of ‘eustress’ that enhances arousal and learning efficiency, rather than a detrimental effect (28, 29). Overall, the current limitations of AI guidance in clinical skills training primarily stem from the one-dimensional nature of its feedback, the potential risk of over-dependence, and the lack of integration with existing curricula. In the future, by integrating multimodal technologies, optimizing teaching model designs, improving evaluation systems, and incorporating ethical considerations, AI is expected to evolve from a practice aid into an intelligent collaborator integrated throughout the teaching process, thereby more comprehensively improving the quality and accessibility of clinical skills training (30, 31).

### 4.1 Limitation

Due to the limited number of included studies and sample sizes, the statistical power of this meta-analysis is relatively constrained, and we cannot entirely exclude the possibility of small-study effects, in which smaller trials may report exaggerated effect estimates. However, our Egger ‘s test did not suggest significant publication bias for the primary outcome. Heterogeneity exists among studies in terms of AI technology types, traditional instruction types, and outcome measurement tools, and the variability in assessment instruments (validated versus self-designed) may have further contributed to this heterogeneity. Although addressed by subgroup analyses and the random-effects model, substantial heterogeneity remained in some subgroups, particularly the LLM subgroup which limits the precision of these estimates. Additionally, due to the specific nature of the intervention formats, many studies could not implement blinding effectively, which may lead to systematic bias. We also restricted our search to English-language publications indexed in PubMed, Embase, and Web of Science, which may have introduced language bias and potentially omitted relevant studies published in other languages or in non-indexed journals. Future research should optimize the dimensionality and contextual adaptability of AI feedback and address these limitations by designing large-scale, standardized RCTs. Finally, regarding the outcome of procedure completion rate, although some studies reported relevant data, a meta-analysis could not be performed for this outcome. Upon in-depth review of the original literature, it was found that these studies ignored the intrinsic correlation among multiple procedures performed by the same learner, leading to overestimated precision of the effect size estimates. Therefore, although the raw data suggest that AI may be beneficial for improving procedural completeness, the statistical robustness of the current evidence is insufficient. Future studies are urgently needed to reassess this outcome using participant-level cluster-adjusted methods, such as generalized estimating equations (GEE) or multilevel models. Concurrently, longitudinal studies should be conducted to explore the long-term impact of AI integration on medical education. Additionally, given the rapid pace of AI advancement, the systems evaluated in the included studies, particularly large language models, may already differ from currently available versions. Our findings should therefore be interpreted as reflecting the effectiveness of AI tools available at the time of the original studies, and ongoing evaluation of newer AI systems is warranted.

## 5 Conclusion

Evidence from this study indicates that in simulation-based training for procedural skills, AI can effectively assume the primary instructional role; in contrast, for training involving complex cognitive skills such as clinical reasoning and assessment, AI should mainly be used to replace inefficient peer practice and provide standardized, high-intensity training, while expert teachers should focus on higher-order clinical judgment, emotional communication, and individualized error correction. Overall, AI presents a major opportunity for quality improvement and large-scale dissemination in medical simulation education, and its optimal application pathway is deep integration with traditional teaching—leveraging their respective strengths—rather than simple substitution.

## Data Availability

The original contributions presented in the study are included in the article/Supplementary material, further inquiries can be directed to the corresponding author.
