# Supplementary material for: Comparison of artificial intelligence assisted training and traditional learning paths in clinical simulation skills training: meta-analysis of randomized controlled trials
Source: Front Med (Lausanne). 2026 Aug 21;13:1915528. doi: 10.3389/fmed.2026.1915528 (PMC13541546; doi:10.3389/fmed.2026.1915528)
Supplement: Supplementary file 1 [file Data_Sheet_1.DOCX]

**PubMed (991)**

((("artificial intelligence"[Title/Abstract] OR "AI"[Title/Abstract] OR "machine learning"[Title/Abstract] OR "deep learning"[Title/Abstract] OR "intelligent tutoring"[Title/Abstract] OR "computer assisted instruction"[Title/Abstract] OR "AI assisted"[Title/Abstract] OR "AI guided"[Title/Abstract] OR "AI based"[Title/Abstract] OR "automated feedback"[Title/Abstract] OR "chatbot"[Title/Abstract] OR "GPT"[Title/Abstract] OR "large language model"[Title/Abstract])) AND (("simulation"[Title/Abstract] OR "simulator"[Title/Abstract] OR "simulated"[Title/Abstract] OR "virtual reality"[Title/Abstract] OR "VR"[Title/Abstract] OR "manikin"[Title/Abstract] OR "mannequin"[Title/Abstract] OR "phantom"[Title/Abstract] OR "standardized patient"[Title/Abstract] OR "simulated patient"[Title/Abstract])) AND (("skill"[Title/Abstract] OR "skills"[Title/Abstract] OR "technical skill"[Title/Abstract] OR "procedural skill"[Title/Abstract] OR "surgical skill"[Title/Abstract] OR "operative performance"[Title/Abstract] OR "psychomotor"[Title/Abstract] OR "dexterity"[Title/Abstract] OR "competence"[Title/Abstract] OR "communication skill"[Title/Abstract] OR "history taking"[Title/Abstract] OR "interview"[Title/Abstract] OR "OSCE"[Title/Abstract] OR "clinical reasoning"[Title/Abstract]))

**Embase (53)**

('artificial intelligence':ti,ab OR 'machine learning':ti,ab OR 'deep learning':ti,ab OR 'intelligent tutoring':ti,ab OR 'AI assisted':ti,ab OR 'AI guided':ti,ab) AND ('simulation':ti,ab OR 'simulator':ti,ab OR 'virtual reality':ti,ab OR 'manikin':ti,ab) AND ('technical skill':ti,ab OR 'procedural skill':ti,ab OR 'surgical skill':ti,ab OR 'operative performance':ti,ab OR 'history taking':ti,ab)

**Web of Science (448)**

TS=((("artificial intelligence" OR "machine learning" OR "deep learning" OR "intelligent tutoring" OR "AI assisted" OR "AI guided") AND ("simulation" OR "simulator" OR "virtual reality" OR "manikin") AND ("technical skill" OR "procedural skill" OR "surgical skill" OR "operative performance" OR communication skill OR history taking)))
