# Supplementary material for: Comparison of artificial intelligence assisted training and traditional learning paths in clinical simulation skills training: meta-analysis of randomized controlled trials
Source: Front Med (Lausanne). 2026 Aug 21;13:1915528. doi: 10.3389/fmed.2026.1915528 (PMC13541546; doi:10.3389/fmed.2026.1915528)
Supplement: Supplementary file 2 [file Table_1.DOCX]

| **Certainty assessment** | | | | | | | **№ of patients** | | **Effect** | | **Certainty** | **Importance** |
| --- | --- | --- | --- | --- | --- | --- | --- | --- | --- | --- | --- | --- |
| **№ of studies** | **Study design** | **Risk of bias** | **Inconsistency** | **Indirectness** | **Imprecision** | **Other considerations** | **[intervention]** | **[comparison]** | **Relative (95% CI)** | **Absolute (95% CI)** |  |  |
| **Overall Clinical Skills Score - Surgical and Invasive Procedural Skills** | | | | | | | | | | | | |
| 6 | randomised trials | not serious | not serious | not serious | serious^a^ | none | 112 | 112 | - | SMD **0.55 SD higher** (0.26 higher to 0.85 higher) | ⨁⨁⨁◯ Moderate^a^ | critical |
| **Overall Clinical Skills Score - Clinical Assessment and Diagnosis Skills** | | | | | | | | | | | | |
| 4 | randomised trials | serious^b^ | very serious^c^ | not serious | very serious^d^ | none | 130 | 132 | - | SMD **1.12 SD higher** (0.15 lower to 2.39 higher) | ⨁◯◯◯ Very low^b,c,d^ | critical |
| **willingness to recommend** | | | | | | | | | | | | |
| 2 | randomised trials | not serious | not serious | serious^e^ | serious^a^ | none | 38 | 38 | - | SMD **1.24 SD higher** (0.75 higher to 1.74 higher) | ⨁⨁◯◯ Low^a,e^ | important |
| **Stress Level** | | | | | | | | | | | | |
| 2 | randomised trials | not serious | not serious | serious^e^ | serious^a^ | none | 66 | 69 | - | SMD **0.57 SD higher** (0.23 higher to 0.92 higher) | ⨁⨁◯◯ Low^a,e^ | important |
| **Procedure Time** | | | | | | | | | | | | |
| 3 | randomised trials | not serious | not serious | not serious | very serious^d^ | none | 40 | 42 | - | SMD **0.43 SD lower** (0.87 lower to 0.02 higher) | ⨁⨁◯◯ Low^d^ | important |

**CI:** confidence interval; **SMD:** standardised mean difference

#### Explanations

a. The sample size included is too small. Downgraded

b. Serious risk of bias since four studies were at high risk of bias. Downgraded.

c. Serious inconsistency since I2 = 95%, Phet <0.00001. Downgraded.

d. The included sample size is small and the confidence interval is wide. Downgraded

e. Serious indirectness since only two trials were available. Downgraded.
